# Human societal development: is it an evolutionary transition in individuality?

**DOI:** 10.1098/rstb.2021.0409

**Published:** 2023-03-13

**Authors:** Yohay Carmel

**Affiliations:** Faculty of Civil and Environmental Engineering, The Technion, Haifa 32000, Israel

**Keywords:** human evolution, cultural evolution, cultural inheritance, social complexity, societal ETI, socio-cultural ETI

## Abstract

An evolutionary transition in individuality (ETI) occurs when a previously independent organism becomes a lower level unit within a higher hierarchical level (for example, cells in an organism, ants in a colony). Using archaeological and historical accounts from the last 12 000 years, I empirically examine the proposition that human society increasingly functions as a higher hierarchical level within which individuals integrate as lower level units. I evaluate human societal development with respect to three criteria that together indicate complexity in biological systems and serve as an operationalization scheme for ETIs: size, inseparability and specialization. The *size* of the largest polity has increased seven orders of magnitude, from hundreds to billions. *Inseparability* became nearly complete since Mesopotamian city-states, following the first appearance of intricate *specialization* (division of labour). *Connectivity* within a polity has increased rapidly during the last few centuries, and particularly within the last few decades. In view of these results, I formulate the following hypothesis: *human society is undergoing an evolutionary transition in individuality,* driven by socio-cultural-technological processes. This proposition requires a detailed theoretical basis and further empirical testing. I propose four predictions derived from the hypothesis that may be used to test it.

This article is part of the theme issue ‘Human socio-cultural evolution in light of evolutionary transitions’.

## 1. Introduction

The emergence of novel levels of individuality is a recurrent theme in the history of life. Biological units that previously existed as independent individuals are incorporated within a higher level of organization, which becomes a new individual [1–4]. For example, multicellular organisms comprise cells whose ancestors were individual unicellular organisms [5,6]. Another example is the transition of individual organisms into a eusocial colony, as illustrated by ants and some bees [7], naked mole rats [8] and marine invertebrates [9]. The whole colony is arguably a single individual, and the ants or bees can be viewed as the mobile equivalents of cells in an organism [7]. These phenomena are termed evolutionary transitions in individuality (ETIs).

Herbert Spencer conceptualized society as an organism, and based much of his sociological theory on this notion [10]. Since the mid-1990s, in view of the increased complexity of human society, some scientists suggested that humans may be undergoing an ETI [11–19] in which human society becomes a new hierarchical level of organization, positioned above the level of the individual person. The organism-like complex society is sometimes referred to as a superorganism [14].

The notion of humans becoming integrated as lower level units within a higher organizational level is supported by the ways in which human societies function like organisms or colonies [20–23]. Social and ecological aspects of humans indicate a fundamental role for the society in our species [24], including division of labour, extra-maternal care and collective decision-making [21]. Human psychology is characterized by behaviours that enable large-scale cooperation between humans [20,22]. Identification with a group is the switch that activates these behaviours, via many mechanisms. These mechanisms include the integration of individuals via communication, unity of action and mechanisms to resolve conflicts of interest in favour of the higher level [20]. Most primate societies are based on individual recognition, where each member in the group identifies other members as a known individual. Humans, by contrast, identify group members even if they are anonymous and unknown by means of markers of identity, such as clothes, accent and hair style [22].

On the other hand, the notion of humans becoming lower level units of a new hierarchical level above the individual is often rejected for various reasons: (i) individual cells and individual organisms that have undergone an ETI to become a multicellular organism or social colony, respectively, are characterized by their relatively low learning capacity and limited behavioural flexibility. By contrast, humans have vast behavioural flexibility; they are fully aware of themselves and their environment, and they care for their freedom, which, it is argued, make them inappropriate candidates for an ETI [25]; (ii) humans are characterized by selfishness, conflict and competition [26], which may offset their capacity for the levels of cooperation required to undergo an ETI; and (iii) cultural evolution has previously favoured human cooperative behaviour. Such favouring diminishes when group size and group interdependence increase to the point that individuals have divided loyalties to multiple groups in many dimensions simultaneously [27].

The debate may be understood as a dialogue of narratives, where more than one view of human society is possible. However, here, I approach it as a scientific question that requires a rigorous quantitative answer. In an earlier study, we operationalized the fuzzy concepts of individuality and transitions to higher organizational levels as a scheme based on measurable criteria [28]. We applied the resulting operationalization scheme (described in some detail below) to various organisms by evaluating our criteria against transitions to multicellular organisms and to eusocial colonies [28]. Here, I apply the same scheme to human societies over time and evaluate the same criteria for a single human society in each time step. The main goal is to compare the course of events in known ETIs with changes occurring in the structure of human societies over time, in order to elucidate whether human societies are undergoing an ETI.

The various stages of ETIs entail an increase in system complexity [1,12,29,30]. Thus, the operationalization scheme was organized around the concept of complexity, based on the assumption that complexity increases during an ETI. This scheme revealed processes that are common across different evolutionary lines and even across different ETI types. The selection criteria required that the scheme's parameters be (i) generable and applicable to any ETI, and (ii) robustly estimable for diverse biological entities as well as various human societies. The resulting operationalization scheme consists of the following three criteria: size, specialization and inseparability:

(i) *size* is the number of lower level units within a higher level entity. It can be the number of cells in an organism, or the number of ants in a colony. In the context of human societies, it is the number of individual members within a single society (a polity: tribe, chiefdom, city-state, nation or empire). Size was found to be correlated with complexity in multicellular organisms [31,32] and in social insect colonies [33]. In the context of human societies, the size of a society affects the rate of its cultural evolution; thus, an increase in societal size corresponds to an increase in the generation of useful novelties [34–36] (but see [37]). Additionally, large societies may undergo specialization more readily than small societies; size could thus be a strong indicator of ETI in humans, similarly to its role in other ETIs;
(ii) *specialization* (also termed ‘division of labour’) relates to the degree of variability in structure and function of the lower level units [3]. As specialization develops, the lower level units (cells of an organism or members of a society) become more dependent on each other and complexity increases [4,6]. In the context of humans, specialization may be estimated roughly as the number of unique occupations within a society. Individuals may switch their professions several times during their lifetime, and specialization is typically reversible. This is still in sharp contrast with a non-specialized society, where all members perform all tasks of daily life; and
(iii) *inseparability* is the incapacity of lower level units (cells, individuals and subgroups) to survive and complete their life cycle separately, that is, independently of the higher level entity. In the context of human society, inseparability is the incapacity of individual persons or small subgroups to separate from a society, to survive, and reproduce entirely isolated from- and wholly independently of other societies. In group-living animals such as wolves, lions, gorilla and chimpanzees, individuals often disperse, and (with a bit of luck) find mates and form new groups, which are entirely independent of, and isolated from their former groups (= full separability). By contrast, in most situations where humans split off from their societies, relations between the new social group and parent society (or other societies) are maintained (for example, Greek colonies on the Mediterranean coasts 2750–2500 yr BP [38]). Such offshoots are not completely isolated and independent, and do not qualify as separable units. Inseparability dictates that individuals can only survive and reproduce as parts of a society [4]. In this sense, the onset of inseparability marks a crucial point in the course of an ETI [6,28].

We used these criteria to evaluate several transitions to multicellular organisms and to social insect colonies [28]. We found that all the inspected ETIs followed a similar sequence of changes in the traits of the novel higher organization level: (i) *size* (at the higher organizational level) increased by many orders of magnitude over the course of the transition process; (ii) *specialization* increased with system size, and presumably also with time, and produced increasing system complexity; and (iii) *inseparability* was a crucial turning point; it appeared early in the process, and it seemed to drive the process forward.

Here, my goal is to apply the same criteria to human societies over time in order to examine whether the temporal evolution of human societies tracks the path of previously identified ETIs, notably the transitions to multicelularity and to eusociality.

## 2. Methods

In order to compare between biological ETIs and the hypothesized socio-cultural human ETI, I apply the above criteria to human societies at various points in time. At any given time, numerous human societies exist simultaneously. Here, only the largest existing social unit (polity) at a given time is assessed with respect to the above criteria. Although size estimates for past societies are necessarily imprecise, an order of magnitude estimate is sufficient for the current purpose. This enhances the robustness of size estimates while still enabling the detection of general trends and patterns.

Societies exist and function beyond polity borders. Networks, organizations and interactions within and between polities are fundamental aspects of society, and enhance its complexity. However, one cannot easily quantify such aspects, and thus this study focuses on polity size as the sole indicator of societal size.

I record points in time at which a major change occurred in at least one of these criteria. Often, I mark and record a point in time at which a specific polity grew to a new order of magnitude for the first time. For example, the Persian Empire 2.5 kyr BP was the first polity to exceed ten million people. I also record times at which large changes occurred in any of the other criteria.

Societies go through periods of development and growth, followed by periods of decline and senescence. To highlight societal transition to a new phase, I record times of societal phase transitions. Ignoring recessions, I only highlight the high-water mark for each increase in societal complexity. Increases and collapses in city population sizes from the Bronze Age onwards have been documented previously [39,40]. However, polity population estimates are difficult to obtain, and the present study may be the first attempt to track changes in polity size during the last 12 millennia. Until around 2000 yr BP, the first polities to set new global size records arose predominantly in southwest Asia. Similar processes that have occurred independently in other parts of the world at later times (see for example [41]) are not accounted for in this study. Comparing independent lines of societal phase transitions is a task for a future study.

## 3. Results

**Figure 1.**
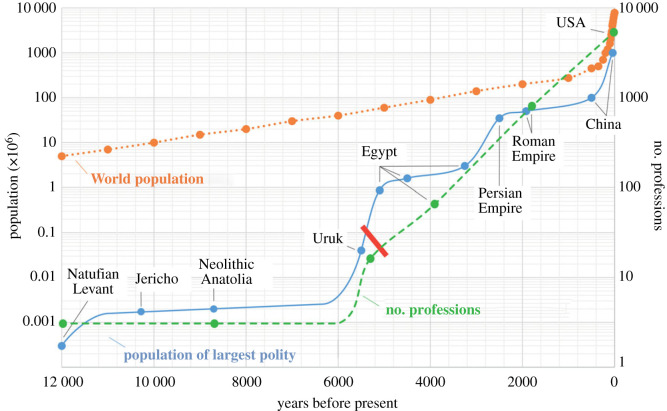
The evolution of human society structure over time, shown in terms of size and specialization (log scales), and inseparability. *Size*: each dot on the blue full line indicates the largest sized polity whose population exceeded that of the preceding named polity by at least one order of magnitude. Global population data derived from ‘Our World in Data’ [42] are shown on the orange dotted line for reference. *Specialization*: each dot on the green dashed line represents the number of professions in a given polity and a given time. *Inseparability*: the red bar indicates the presumed first appearance of inseparability. See the electronic supplementary material, table S1 for numeric values of these data points and references to their sources. (Online version in colour.)

The structural evolution of human society is quantified using the criteria described above (figure 1).

*Initial social structure*. Humans were hunter–gatherers at least since the lower Palaeolithic [43]. Palaeolithic people lived in bands of about 25–40 people [44]. Several bands typically belonged to a tribe, a higher level social construct. Individuals moved frequently from one band to another within the same tribe [45], which thus served as a large genetic pool for its population [22]. Archaeological findings indicate that band societies were materialistically egalitarian; division of labour was probably restricted to hunting versus gathering, and possibly to some toolmaking [46,47]. Larger, semi-sedentary, non-egalitarian societies also existed, particularly in provident areas [48]. During the Early- and Middle Epipaleolithic, 24–15 kyr BP, some changes in the lifestyle of bands in southwest Asia took place, as expressed in novel archaeological findings from these periods. These changes included the appearance of the first permanent settlements [49,50] as well as harvesting of wild plants (grasses, barley and wheat) and processing of their seeds [51,52]. However, these changes did not modify the social structure of the tribe (as expressed by the indicators evaluated here). The few permanent settlements were embedded within a society of nomadic, egalitarian bands, with no indication of hierarchy or division of labour [53].

*Size*. The first fundamental change in the size and structure of the social unit took place in the Late Epipaleolithic period (15–12 kyr BP) in the Natufian Levant. Most Natufian sites were permanent settlements [54]. Population size in the larger sites was 100–200 people [55], larger than typical band sizes in previous eras. These sites were connected by large-scale social networks, probably encompassing 10–30 settlements over the entire Natufian Levant [56], as indicated by extensive exchanges of goods [53], similar architecture [57] and similar burial patterns [58]. The total Natufian population in this network consisted of a few thousands for the entire Levant [56], which is an order of magnitude larger than the estimated sizes of tribes of previous periods.

During the Neolithic, with the spread of agriculture, another 10-fold increase in population size occurred in the Levant [59]. In Jericho, a massive tower and walls were constructed, dated 10.3 kyr BP [60], indicating large population size, possibly in the range of 2000–3000, although smaller numbers were also suggested [61]. The ‘town’ of Çatalhöyük in Anatolia hosted at least 3500 people at its peak, 8.7–8.5 kyr BP [62]. The strength of Neolithic social networks is also indicated by the Göbekli Tepe structures, dated *ca* 11.5 kyr BP, with the cooperation of hundreds of people required to erect the massive pillars [63]. Thus, within seven millennia, social unit size increased from dozens to thousands. However, a larger and more rapid increase occurred in Mesopotamia within a 1400-year period (5.8–4.4 kyr BP, see below). This revolution coincided with the appearance of the first urban centres and with the appearance of writing.

The emergence of the first city-states in Mesopotamia at approximately 5.8 kyr BP [64] marked an increase in societal size to 10^4^. The most prominent city-state was Uruk, whose population at its peak was approximately 40 000 people [65], with approximately 90 000 inhabitants including its hinterland [66]. Soon, another order of magnitude increase in polity size followed, with the first unification of Egypt under a single king around 5.1 kyr BP [67], when the total Egyptian population was about 0.9 million [68]. A few centuries later, by around 4.5 kyr BP, that population had risen to about 1.6 million [68]. The Akkadian Empire (4.3 kyr BP) controlled a population that was probably much larger than one million, but exact demographic data are unavailable.

The next leap in maximum polity size occurred approximately 2.5 kyr BP, when the first Persian (Achaemenid) empire totalled 17–35 million people [69], making it the first polity to surpass 10^7^. Then, for over 2000 years, the largest polities—the Roman Empire [69], the Chinese Empire [70] and the Islamic Caliphates—fluctuated in the range 10–100 million people. By 500 yr BP (AD 1500), China's population exceeded 10^8^ [70] and crossed 10^9^ by 1982 [71].

An alternative view of the largest societal entity considers the global network as a single coherent and interconnected social and economic unit. This network now includes essentially the full global population, approximately 7.9 × 10^9^ [72]. This makes human society comparable in size to the largest social insect colonies, but smaller than the cell count (10^10^–10^15^) of most multicellular organisms [28].

*Specialization*. Division of labour may have existed in some Palaeolithic societies, as women were mostly gatherers whereas men were mostly hunters [46,47]. Tool production may have involved some level of individual specialization in sophisticated lithic cultures, such as the Kebaran [53]. Yet, the number of roles, or ‘professions’ in those societies was in the range of 2–4. Even during the Late Epipaleolithic and Neolithic periods (15–6 kyr BP), the growing sedentary settlements in the Levant and Eastern Anatolia showed no indication of increase in specialization [56,73]. The first intricate division of labour appears in the early Uruk Period (approx. 5.8–5.1 kyr BP). There is firm evidence of specific town areas dedicated to specific crafts, including pottery, textile and metals [65]. The large Warka Vase, a pioneering piece of art dated approximately 5.1 kyr BP, shows officers, clerks and weavers [74]. There were also farmers, temple workers, rulers, merchants, soldiers, temple builders, mosaic makers and others [65]. Taken together, the first city-states had more than ten distinct crafts/professions. Possibly, the appearance of a central governance promoted this specialization. This revolutionary social transformation probably also marked a transition from egalitarian to coercive societies; a plausible mechanism for such a transition was proposed by [75]. In ancient Egypt approximately 3.9 kyr BP, the ‘Instruction of Dua-Khety’ details 20 trades [76]; texts from that period mention additional professions, making their total number greater than 60 [77,78]. In the entire Roman Empire, there were several hundred separate occupations [79,80]. Current lists of professions are typically in the range of thousands of unique occupations; for example, the number of occupations listed by the US Bureau of Labour Statistics [81] is 5412. Additional examples are provided in the electronic supplementary material. City population size is a strong predictor of diversity with respect to professions in current societies [82] as well as in the ancient world [79]. Consequently, population size and division of labour are correlated in human societies, similarly to the situation in multicellular organisms [83] and insect colonies [84,85].

*Inseparability*. This is the incapacity of a subset of a society's members to separate entirely from their social network and survive wholly independently. As society becomes more complex, members of a society specialize in specific functions, and single individuals become more and more dependent on numerous other societal members. No single person or even small subgroup would thus have the complete set of expertise required for the survival of a new self-sufficient, independent group. In hunter–gatherer bands, an event where one or a few band members leave and start a new band of their own is conceivable, albeit rare [22]. Most band members participate in most band functions and therefore possess most of the skills needed for a new start; with a bit of luck, a new independent band is established [22]. Similar events may also have happened during the first phases of sedentary societies, including the Epipaleolithic and Neolithic, as they were probably egalitarian societies with little specialization [56]. Budding of new independent societies established by a small group of farmers who leave their land and migrate beyond the reach of existing societies was therefore still possible (even if risky and uncommon). Among the few known examples of such events is the settlement of Rapa Nui (Easter Island) by Polynesians between the twelfth and thirteenth centuries CE [86]. Polynesian society was constrained by land limitation [87] that was arguably more severe than land limitations faced by city-states located on larger land masses. A total lack of data on such events in the distant past makes it impossible to quantify inseparability; it is, however, reasonable to assume that it was incomplete in hunter–gatherer tribes. Central control and a strong division of labour in urban societies, such as occurred in the Mesopotamian city-states of the sixth millennium BP onward, probably drastically reduced the frequency of separation events from such areas. In the following millennia, interdependence between different sectors in society continued to increase in city-states and, subsequently, nation states, largely owing to rising specialization, thus further diminishing the probability of separation events. Consequently, it is reasonable to assign nearly complete inseparability to all city-states, kingdoms and nations, ever since urbanization first took place.

## 4. Discussion

### (a) Hypothesis

This study consists of a survey of the structure of human societies over time, focusing on features indicative of a possible ETI. The results reveal increasing complexity (in terms of size, specialization and inseparability) of human societies over time, resembling (but not identical to) trends in ETIs of multicellular organisms and social insects [28]. Considering these results, I suggest the following hypothesis: *human society is undergoing a socio-cultural transition in individuality*. The disparity between human societal transformation and biological ETIs (see below) casts doubts on framing the former as ETI [88]. The three abovementioned criteria are important indications of an ETI, but each of them separately could arise from non-ETI processes. The proposed predictions stemming from the socio-cultural ETI hypothesis (see below), if tested and proven positive, could provide additional support for the hypothesis. Further theoretical work is needed to clarify and fine-tune the hypothesis, and possibly propose a detailed mechanism.

Two groups have recently proposed similar hypotheses. Andersson and colleagues [11,15] date a societal ETI much earlier than is proposed here, to the emergence of the genus *Homo* 2.5–1.8 million yr BP. They maintain that the new higher level of selection that resulted from this ETI was a biological-social-technological entity termed a ‘sociont’ [11,15], which consisted of the unique inherited cultural traditions associated with a specific clan or tribe. Waring & Wood [13] discuss the pre-emption of cultural over biological evolution in humans and maintain that, in human evolution, adaptive information gradually shifts from the genetic-inheritance system to the cultural inheritance system. Common to both propositions, as well as to the general hypothesis of this study, is the notion that cultural evolution drives human societies into a socio-cultural ETI.

This notion provokes diverse reactions, ranging from resentment to contentment. Some of the discontent may stem from a world-view of humans as independent intelligent entities [25] and may not easily give up their individuality; the present hypothesis challenges this view. For biologists, the notion of a socio-cultural ETI is challenging for another reason, though. The societal transition differs substantially from biological ETIs in at least two fundamental aspects: (i) in addition to humans, intangible entities such as memes [89], cultural traditions [11], and possibly AI elements [90] may come to play important roles; conceptualizing such a transition involves interactions between biological- and non-biological elements; and (ii) unlike biological entities, human societal structure does not fit the standard framework of a single higher level entity enveloping its lower level units. Each individual may have several affinities to several nested- and non-nested higher level entities simultaneously.

### (b) Comparing the socio-cultural evolutionary transition in individuality with biological evolutionary transition in individualities

Application of the ETI operationalization scheme described above to human societies over the last 12 000 years revealed changes in all three parameters.

*Size*. The size of the largest social unit or polity increased by seven orders of magnitude (approx. 10 million-fold) during this period. This increase was nonlinear, with the largest polity growing dramatically from thousands to millions over the 1300 years from 5.8 to 4.5 kyr BP, but taking another 4400 years to increase from millions to billions. Similarly, in all ETIs, the size of the higher level entity increased by several orders of magnitude [28].

*Specialization*. There was very little division of labour in hunter–gatherer societies and early farmer societies. A radical and swift change occurred in Mesopotamian city-states 5.8–5.1 kyr BP, with the emergence of more than ten types of professions. Since then, the number of professions and occupations increased to dozens, then hundreds, and most recently to thousands, indicating a consistent increase in specialization.

Population size and specialization are correlated in human societies, similarly to the situation in multicellular organisms and insect colonies. However, differences between human societies and biological ETIs occur with respect to the specific type of specialization. The initial specialization in biological ETIs is reproductive [28]. In earliest multicellular organisms, the first differentiation between cell types is between somatic- and reproductive cells [5,6]; similarly, the first differentiation in eusocial insect communities is between queen and sterile workers [7]. These specializations occur in functional units sized in the hundreds, whereas other specializations occur much later in the ETI process, in entities larger than millions [28]. By contrast, the results of this study show that non-reproductive specializations appear in all human societies larger than a few thousand individuals, whereas reproductive specialization has not occurred even in a society of billions.

This difference stems from the essential difference between biological ETIs and the hypothesized socio-cultural ETI. Jablonka [2] proposed an interesting insight: biological ETIs proceed through modification of the genotype and thus require physical extinction of lower level units and differential reproduction of the organism/social colony. By contrast, the socio-cultural ETI need not involve the physical extinction of individuals or differential reproduction. Societies may evolve, and their complexity may increase, based on mere changes in the behaviour of their members [2]. Thus, reproductive specialization is not required for socio-cultural ETI. Moreover, these differences may reflect the difference between the genetic-inheritance system of the biological ETIs, as opposed to the cultural inheritance system behind the hypothesized socio-cultural ETI [2,13]. In socio-cultural ETI, information propagation is not only vertical (as in genetic transmission in biological evolution), but also horizontal (between non-kin society members) [13,91]. This implies a much faster, yet less accurate, information transmission, resulting in rapid and divergent evolution [14,92]. In biological evolution, the progress of ETIs may take millions of years; by contrast, socio-cultural ETI advances much faster. In genetic-inheritance systems, specialization is first and foremost reproductive, whereas in socio-cultural ETI, specialization is first and solely non-reproductive.

Specialization drove the structure of human societies in an ETI direction. Since its first appearance between 5 and 6 kyr BP, the division of labour has steadily intensified, yielding increasingly complex societies. Several subsystems have emerged within these societies, somewhat similar to subsystems within an organism [11]. Transportation systems in major urban areas have much in common with circulation systems in an organism. Electronic communication can be likened to a nervous system. Legal and policing systems can be viewed as crude forms of immune systems. The systems mentioned above are much simpler and less effective than their biological equivalents within an organism. However, considering the speed at which they developed relative to organismal evolutionary timescales, their mere existence suggests a fundamental potential of cultural-social-technological evolution to transform societal structure.

Polities emerge, grow and collapse, often rapidly. This serves as an indication that the hypothesized transition presumed here is in its embryonic phase. Societies could be likened to primordial organisms, where primitive and partial integration dictates short life-span.

*Inseparability*. Once elaborate specialization emerges in a society, single individuals and small subgroups lack the knowledge required to construct a new society on their own. Since the first profound specialization of human societies in the sixth millennium BP, barriers to separation rose ever higher, rendering city-states, kingdoms and nations effectively inseparable.

Inseparability, a causal driver of biological ETIs [28], may have contributed also to the socio-cultural ETI. In a society with complete inseparability, the fate of an individual is completely dependent on the fate of its society. As a result, selective pressures on individuals decrease and selective pressures on the society (tribe/band) rise. The society as a whole becomes the primary unit of selection (in the sense of [1]). Some societies may die out, or may be absorbed into stronger societies [93]. These selective pressures increase the mean size of the society, enhance its efficiency and promote technological innovations. However, in addition to competition, the relations between societies consist also of mutualism (for example, inter-polity trade and treaties, as well as cultural interactions). For human societies, large-scale exchange arose as early as the Neolithic period, 12 kyr BP. Exchange distances and material diversity increased with time. Currently, strong and dense economic networks (all-to-all) link nations and corporations, whose interdependence highlights their role as semi-independent units, or as cogs in a loose global societal machinery [72].

In addition to these criteria, it was suggested that increased connectivity between lower level units (here, humans in a society) may indicate increased integration of the emerging higher level organism [28,94]. Connectivity is difficult to quantify in organisms [28] as well as within societies; it seems, however, that connectivity between society members has increased in recent centuries, and particularly since the invention of communication technologies such as the telephone, internet, etc.

### (c) Predictions and future testing of the hypothesis

To qualify as a scientific proposition, the presented hypothesis should allow researchers to make well-defined, testable predictions. Based on the hypothesis, I predict that several directional changes accompany increasing social complexity over time. Three such traits, namely, size, specialization and inseparability, were evaluated above. These three traits are not mere ‘indications’ of ETI; they are considered to be in the core of the process and possibly its driving forces, thus supporting its framing as a ETI. Based on the abovementioned hypothesis, it is predicted that four additional traits change with increasing social complexity:

(i) the level of *regulation and control* that society exerts over its members should increase. ETIs always begin with independent organisms that combine into a novel collective entity; the newly emerging entity initially has very little control over its constituents, as control mechanisms have not yet developed. As the transition progresses, control by the higher level entity over its lower level units may have strong selective advantages compared with entities that lack such central control. Hence, I predict that, as the socio-cultural ETI progresses, societal control over its members will rise correspondingly. The reverse, reduced control, is expected to emerge not gradually, but rather suddenly, propelled by a collapse, such as may occur following take-over by other societies, internal splintering, or severe natural disasters. One could evaluate increased control using various proxies, such as the number of new regulations per year;
(ii) the extent to which the *individual's basic needs* (food, health and personal safety) are met by society should increase in terms of the number of different needs covered and/or the degree to which they are fulfilled. One indirect measure of this is fluctuations in the number of distinct ministries and departments in governments over time;
(iii) the proportion of *conflicts between an individual and society* that are settled in favour of society should increase. This could be estimated by surveying the outcome of ‘state versus individual *x*’ trials; and
(iv) the strength of the relationship between climate and population size should *diminish*, because as society becomes more complex, its care for its members rises, and therefore their fate is less impacted by environmental conditions and more by how the society fares as a whole.

The framing of human societal change as an ETI is challenging. The arena moves from the biological to the cultural domain, from tangible organisms and genes to intangible social and cultural entities. Variations on this theme were proposed few times in the past two decades [11–19] but this notion remains largely unknown and poorly studied. The concept as presented here is general and lacks theoretical detail. However, viewing human societies in the context of ETI may revise our conceptions of the individual-society relations and therefore calls for deeper examinations.

### (d) Two views of society and individuals

The notion that human societies have become increasingly complex is widely agreed upon [64,95,96]. The implications of this process for humans as individuals are contested. A prevailing view regards human society as empowering the individual. As societies grow and develop ever-stronger control, the situation of the individual improves in terms of personal safety, assured meeting of physical needs (water, food, clothes and housing), enhanced freedom, reduced war and better education [97]. In the opposing view, society increasingly controls and minimizes the autonomy of its individual members in a process termed by McShea ‘machinification’ [30]. This view is particularly prominent in literature and the arts, notably in the works of Franz Kafka, George Orwell, Aldous Huxley, Kurt Vonnegut and Charlie Chaplin, but also in academic publications [98]. Recently, even liberal and democratic states have evinced symptoms of rising authoritarianism, that is, increased societal control over the individual [99–101].

Interestingly, both views are backed by voluminous evidence. On the one hand, societies protect their members from mishaps arising from the external environment. The proportion of humans directly affected by murders, famine, and even war decreases with time [97]. Governments are able to protect their citizens more effectively than in the past, even in the case of major societal perturbations such as the current COVID pandemic. Similarly, cells gain increased protection when incorporated into an emerging multicellular organism [6,84]. On the other hand, rising control by governments over their citizens is obvious [101], even if far from universally welcomed. These two contrasting trends may actually be complementary expressions of the same process: society becomes more cohesive, centralized and effective. Society increasingly meets its members' needs, while simultaneously exerting tighter control over them at the group and individual levels. Interestingly, both views are consistent with the hypothesis that human society is currently undergoing a socio-cultural ETI.

## Data Availability

All data mentioned in this manuscript, including references to data sources, are available on the supplementary material attached within this submission [102].
